# Multidisciplinary blended learning to build a breast cancer specialist career: survey on the perspective of the first 2 cohorts of the ESO-ULM Certificate of Competence in Breast cancer (CCB)

**DOI:** 10.1186/s12909-022-03414-7

**Published:** 2022-05-05

**Authors:** Francesco Meani, Tibor Kovacs, Wiebke Wandschneider, Alberto Costa, Olivia Pagani

**Affiliations:** 1 Centro di Senologia della Svizzera Italiana (CSSI), Ente Ospedaliero Cantonale (EOC), Lugano, Switzerland; 2 Department of Obstetrics and Gynaecology, Ospedale Regionale di Lugano, Ente Ospedaliero Cantonale (EOC), Lugano, Switzerland; 3 Breast Unit, Guy’s and St Thomas’ NHS Foundation Trust, London, UK; 4 Breast Institute, Jiahui International Hospital, Shanghai, China; 5 ESO- European School of Oncology, Milan, Italy; 6 Interdisciplinary Cancer Service Hopital Rivera-Chablis Rennaz, Vaud, Geneva University Hospital, Geneva, Switzerland; 7 Faculty of Biomedical Sciences, Università della Svizzera Italiana, Lugano, Switzerland

## Abstract

**Background:**

Breast Cancer (BC) specialists need to acquire comprehensive knowledge, covering their own specialty and principles of related disciplines. Blended learning, the integration of online and face-to-face learning, is becoming more and more important in academic education and has added value during pandemics which limit face-to-face learning and residential training.

In this context, the ESO-ULM Certificate of Competence in Breast Cancer (CCB) provides postgraduate multidisciplinary education and delivers an academic postgraduate title.

The aim of this work is to investigate the degree of satisfaction of 42 participants to the first two editions of the programme and to assess if attending the programme entailed any professional gain.

**Methods:**

An ad-hoc questionnaire was developed exploring 4 areas: participants’ characteristics, administrative aspects, CCB Program syllabus and design, professional impact.

**Results:**

The program was attractive for specialists of different disciplines from all over the world: > 90% of responders appreciated the curriculum set up and the quality of the teaching.

Despite 64% of responders changed their clinical practice, only 33% could implement institutional changes. One third of the participants activated a collaboration with other colleagues and 64% used the CCB as a trigger to take part in other educational activities.

Only 12% of the participants had the opportunity, after CCB, to visit other BC Units or to be involved in international research projects.

More than half of the attendees profited from attending CCB in terms of promotions (16.7%), change of working institution (9.5%) or development of a more structured educational program at their home institutions (28.6%).

**Conclusions:**

Results provide interesting and stimulating considerations on the expectations and needs of training physicians and on what modern educational tools and formats can achieve. This paper can provide useful information to navigate through what the post-graduate training market is currently offering to develop a specific curriculum in modern multidisciplinary BC care but might not be applicable to other fields of multidisciplinary oncology.

**Supplementary Information:**

The online version contains supplementary material available at 10.1186/s12909-022-03414-7.

## Background

Breast cancer (BC) management is a complex and rapidly evolving discipline which can no longer rely on generic, single specialty training. Evidence has shown that multidisciplinary specialized care improves patient outcomes [1]: according to the 2006 European Parliament resolution, BC diagnosis and treatment should be provided in certified Breast Units, equipped with qualified, adequately trained and dedicated health-professionals [2]. BC specialists need to acquire a comprehensive knowledge, covering not only their own specialty but also the principles of related disciplines, to build a dedicated multidisciplinary curriculum (CV) [3]. Despite the wide consensus to develop quality standards, there is still great variability and lack of standardization of BC care across Europe [4], partly due to heterogeneous training systems across countries [5]. The gap between the recommended BC care/training and actual practice is even bigger in low-middle-income countries where resource limitations greatly impact the provision of quality-controlled services and patients’ outcomes [6].

Medical education is changing, and modern technology enables the learner to access didactic material, at any time, and at any location [7]. Blended learning is the integration of online and face-to-face learning and is becoming more and more important in academic education [8–11]. This approach is of added value during pandemics which limit face to face learning and residential training [12, 13].

In 2014, the European School of Oncology (ESO), in co-operation with the Ulm University, developed a structured course, named “Certificate of Competence in Breast Cancer (CCB)”. It aims at providing BC specialists with multidisciplinary education and delivering an academic postgraduate title in the field. The Curriculum, built with the contribution of internationally recognized BC clinicians and scientists, provides, and enhances, clinical and scientific competencies, recognized by an academic postgraduate title. CCB provides a total of 381 hours of comprehensive learning (75 in-person and 306 of distance learning) corresponding to a workload of 13 European Credit Transfer and Accumulation System Points (ECTS) accredited by the Ulm University. The Program includes five modules and three attendance seminars with an overall duration of 13 months (Objectives and concept - European School of Oncology (eso.net ).

The CCB program, with its academic endorsement, meets all the requirements to be part of the BRESO- Breast Surgical Oncology project, created in 2018 to enhance and harmonize breast surgery training across Europe [14, 15].

CCB has now reached its fourth edition. Forty-two specialists were admitted to the first two editions of the program and have passed the final examination at ULM University, obtaining the academic Certificate of Competence (CCB). Twenty-five health professionals will participate in the fourth round (2021-2022).

The acquisition of a multidisciplinary, highly specialized, structured CV, with academic qualification, should also enhance professional carrier opportunities.

The aim of this work is both to investigate the degree of satisfaction of participants to the first two CCB editions and to assess if any professional gain, both on a personal and institutional level, was achieved by attending the CCB program. With this work we hope to aid trainees, seeking to develop a specific curriculum in multidisciplinary management of BC, to navigate through the offer of the market and make a more informed choice according to their career expectations.

## Methods

An ad-hoc questionnaire was developed by a specialist task force composed by a senior Breast Surgeon, a senior Oncologist, one young Gynecologist involved in post-graduate training in BC surgery, one professor from the Faculty of Communication at USI (Università della Svizzera Italiana) and one ESO coordinator of post graduate training programs. The poll was then piloted on a different group of breast surgeons, oncologists, trainees, and administrative personnel to ensure content validity and usability. Minor modifications were made based on feedback: the definitive survey explored 4 domains:

1. Participants’ background information (demographics, field of specialty, level of training/clinical experience, reasons for enrolling, etc.)
2. Administrative aspects of the CCB Program and application
3. CCB Program syllabus and design
4. CCB Program outcome and professional impact

At the very end of the questionnaire, participants were asked to rate the overall quality of the course and were given the opportunity, by means of two open questions, to provide the organizers with personal suggestions to improve future editions.

The final version of the survey was reviewed and approved by all members of the task force and then published online, using the GOOGLE Forms platform.

All 42 participants from the first two cohorts of the CCB Program were invited to participate by an e-mail sent out from the central ESO office, which contained a link to the questionnaire.

The survey was available online for 8 weeks, and two email reminders were sent out during this period, at the end of which a response rate of 100% was achieved thanks to the notable commitment of all CCB participants. The full questionnaire is available on Additional file 1: Appendix A.

After the survey was closed, spreadsheet data were exported for analysis: responses to the questions were extracted and summarized.

## Results

### Participants’ background information

#### Demographics

The cohort included 42 physicians (Table 1), from 4 continents and 27 countries, the most represented being Brazil (4 - 9.5%), Italy (3 - 7.1%) Switzerland (3 – 7.1%) and South Africa (3 - 7.1%*).* Interestingly a significant share (6 - 14.2%) was from low-middle income countries: 2 from Egypt, 2 from Georgia and 2 from Pakistan. All invited health professionals answered the survey.

**Table 1:** participant’s characteristics

	n. (%)
Number of participants	42
Country of Employment:	
High income	36 (86)
Low-middle income	6 (14)
Age, years, median (25th-75th percentile)	40.8
Gender	
Female	27 (64)
Male	15 (36)
Educational level, n (%)	
Head of department/Professor	11 (26)
Consultants/Attending Physicians	17 (41)
In training (Resident, fellow, PhD-stud., post-doc)	14 (33)
Specialty, n (%)	
Medical oncologist	29 (69)
Gynecologist	7 (17)
Radiation oncologist	5 (12)
General surgeon	1 (2)

Mean age of the participants was 40.8 years (ranging from 31 to 61); 27 (64%) were females and 15 (36%) were males. All specialties were represented: 29 (69%) participants were medical oncologists, 7 (17%) gynecologists, 5 (12%) radiation oncologists, and 1 (2%) general surgeon. Eighteen (42.9%) respondents worked in a university hospital, 15 (35.7%) in a non-teaching public hospital and the remaining 9 (21%) in private clinics.

The degree of training/professional position at time of enrollment varied significantly, ranging from post-doc/PhD students (3 - 7.1%) to chiefs of department (8 - 19.0%) and Professors (3 - 7.1%); consultants were the largest group (17 - 40.4%), the remaining being residents and fellows (11 - 26.2%).

#### Reasons for enrolling

When asked about the main reason for applying to the CCB Program (more than one answer was allowed together with an open answer to include reasons not listed in the available options) most of the participants provided 2 answers, the most frequent being: 1) the need for up-to-date comprehensive knowledge in BC, 2) the search for a model of training focusing on multidisciplinary management.

Other reasons included the opportunity to develop new international collaborations for clinical and research projects (networking and learning about BC treatment in other countries) and, not least, the wish to improve their CV to boost a career advancement.

### Administrative aspects of the CCB program

This section of the survey aimed to explore participant’s opinion on the CCB administrative procedures. Overall, the vast majority (39–93%) of participants agreed that website information (www.ESO.net) was informative and precise, the application procedure was clear, easy to follow and reasonably structured in terms of time allocation.

Over 95% (*n* = 40) of responders believe that selection criteria for admission, based on personal CV and specific BC experience were correct, about 20% (*n* = 8) considered that final exclusion/inclusion decisions were not fully transparent.

Thirty percent (*n* = 13) of responders thought the application fee (5400 Euros) was somehow too expensive, despite a discounted fee available to participants from low, lower middle and upper middle-income economies. Interestingly, most participants were either from low-middle income countries (according to the Organization for Economic Co-operation and Development-OECD) and/or did not have a permanently paid professional position (students or residents), three outliers had stable professional positions in high income countries.

### Program design and syllabus

Most of the surveyed professionals agreed that the curriculum is well balanced, covering all aspects of multidisciplinary management of BC (37-88%), in a very updated and detailed manner (40-95%).

Two aspects the participants would like to be improved are the opportunities for networking (13-30%) and the implementation of collaborative research projects among participants and possibly among home institutions (18-43%).

When specifically asked for suggestions to improve the program structure (more than one answer was allowed), five aspects were highlighted:

1. To include periods of structured observer-ship (30-71%)
2. To increase the time allocated to multidisciplinary discussions during seminars (27-64%)
3. To increase the time spent in the hospital during seminar 3 in Ulm (24-57%)
4. To increase the focus on loco-regional treatments (17-40.5%)
5. To increase the opportunities of clinical research training (15-35%)

When looking specifically at the possibility to increase the knowledge on surgical and loco-regional treatments, the cohort felt divided. The responses have been variable: 64% (*n* = 27) were definitely in favor, 12% (*n* = 5) undecided, 24% (*n* = 10) against. It is of note that 20 (48%) medical oncologists and 2 (5%) radiation oncologists were in favor, underpinning the concept that one aim of BC professionals is to widen the horizons of their knowledge, in line with the goal to improve multidisciplinary skills.

### Program outcome and professional yield

#### Personal yield

When investigating personal professional benefits, 64% of responders (*n* = 27) declared to have implemented a significant change of BC management in their clinical practice consequently of what they learnt through the CCB Program.

In particular, they have developed better awareness and active participation in decision making processes during the multidisciplinary meetings (MDMs); some felt to have improved team working skills and gained capacity to better organize teaching sessions for students and fellows.

#### Institutional yield

When shifting the attention to the institutional level, the landscape changed: 67% of respondents (*n* = 28), upon returning to clinical routine, did not report any significant change in clinical guidelines or in the organization of the Breast Center at their home institution. Among the minority (33%, *n* = 14) who were able to implement some changes, update and innovations in internal protocols and guidelines are reported. Development or improved efficacy of MDMs is also a goal worth to mention. One responder (2%) stated to have set up a brand-new department dedicated to BC care, using the experience gained from the course and from his/her international colleagues.

#### Networking (personal or formal agreement with other institutions)

Opportunities for networking and experience sharing are well among the objectives of an international program of postgraduate advanced studies.

This domain was investigated through two specific questions about the activation of collaborative scientific projects among individuals or institutions.

On an individual level, one third (33%, *n* = 14) of the participants were able to activate a collaboration with other colleagues which yielded two peer reviewed publications, several new research ideas, and international connections for networking and mentoring opportunities. On the other hand, 66% (*n* = 28) of the CCB fellows thought they had not achieved a significant networking advantage from attending the program, at least at the time of the survey.

When looking at the Institutional level, 5 participants (12%) had the opportunity for visiting other BC Units in foreign countries, for example through the ESO fellowship program. One responder was able to get his home institution on board a multicentric international research project with Breast Units from London and Paris.

#### Concrete outputs

Seventeen responders (40.5%) took part in a publication on a peer-reviewed international journal, 2 (4.8%) co-authored a book chapter, 3 students (16.7%) took advantage of the CCB participation to develop their doctoral thesis and 7 (16.7%) have arranged an exchange plan for themselves or other students. One (2%) participant, originally from a low-middle income country, but employed in the USA, was invited to participate in the panel of experts to review the ASCO 2019 Meeting Educational Book, together with several renowned international BC experts.

On the other hand, 3 responders (31%) said they had not gained any specific professional output from the CCB other than general multidisciplinary knowledge update.

#### Professional gain/advancement

The main professional gain acquired from the certificate was related to multidisciplinary management of BC: 95% of responders (*n* = 40) reported an increased awareness of the relevance of, which paralleled their enhanced professional self-confidence. Their participation to MDMs discussions became more active and their clinical decisions established according to up-to-date knowledge. Finally, the survey scouted on specific changes in professional career that, directly or indirectly, derived from attending the Certificate: 7 participants (16.7%) reported a promotion consequent to the additional qualification achieved; 4 responders (9.5%), decided to change working institution; 12 (28.6%) implemented a new and better organized educational program at their home institutions.

The majority (64%, *n* = 27) of the attendees, used the CCB as a springboard to take part to other educational activities, e.g., preceptorships or formally organized Breast Cancer Fellowship programs.

#### What to keep and what to improve after CCB

The last two questions of the survey pertained to what the participants liked and disliked the most.

#### Thumbs up

focus on multidisciplinary care; international environment and networking opportunities; participation in international top-level conferences; self-managed online lectures covered by an outstanding faculty; access to relevant and updated literature. The program was well designed and structured to deliver, within a relatively short period of time, a comprehensive picture of modern multidisciplinary BC management, with in depth analysis of hot topics in the field. The general organization was appreciated, being suitable not only for students in training but also for busy physicians.

#### Thumbs down

the program seems a little skewed toward the medical oncology side; somehow lacking in depth discussion on surgical and loco-regional treatments. A significant share of participants would have liked to be offered some more opportunities for practical activities and/or observerships during the seminars. Finally the online system did not encourage interaction enough, resulting in a lack of discussion among participants, as well as with the faculty.

## Discussion

BC care, one of the paradigms of multimodal care, is increasingly complex and requires a broad knowledge of several multidisciplinary treatment strategies, that only a dedicated team of health professionals can deliver. The quality of medical care is therefore strictly linked to the quality of training provided to medical professionals. Quality-improving training programs in oncology have been implemented in a variety of cancer delivery settings in the U.S. and helped maintain the medical competence in practice [16, 17].

Currently, no standardized training in oncology and specifically in BC treatment, whether for surgical or medical disciplines, exists outside the U.S. Consequently, among both, specialists dedicated to BC care and young doctors, there is a sheer desire for up-to-date multidisciplinary training. In this setting, the ESMO/ASCO Recommendations for a Global Curriculum in Medical Oncology have been conceived with the goal of defining standards in guiding the training of medical oncologists worldwide and to ensure that all patients have an equal chance of receiving treatment from well-trained physicians.

Similarly, on the surgical side, some of the major European societies involved in BC surgical training, research, education, and advocacy recently developed the *BRESO (Breast Surgical Oncology Project)*: a widely endorsed attempt to enhance and harmonize breast surgery training across Europe by means of a structured, high-level, multidisciplinary training and certification [14, 15].

E-learning programs can play a significant role facilitating distance learning, as they may overcome some of the difficulties seen with traditional learning and training by allowing flexibility in time, place, and pace, for both the clinically working trainee and educator. Several models can be adopted [8] and it is calculated that > 80% of U.S. doctoral/research institutions have some form of online offering [7], either courses or full programs.

The ESO-Ulm Certificate of Competence in BC is one of the very few academic programs specifically designed to meet the needs of professionals dealing with BC and has been incorporated and recognized by the BRESO certification. CCB has been designed to cover and fulfill knowledge needs and improve career trajectories of professionals coming from all specialties involved in BC management, at any level of professional experience, from fellows to chiefs of departments.

The current survey, designed by the participants of the third CCB cohort, showed the program was attractive for specialists of different disciplines from all over the world: > 90% (*n* = 38) of responders appreciated the curriculum set up and the quality of the teaching. Nevertheless, several practical suggestions for improvements were made. Among them the most popular were: including periods of structured observer-ship (30-71%); increasing the number of multidisciplinary discussions (27-64%) and the time spent in the hospital during live seminars (24-57%); focusing more on loco-regional treatments (17-40.5%). The last proposal was supported not only by surgeons, radiation oncologists and gynaecologists but also by 69% (*n* = 20 out of 29) of the medical oncologists, demonstrating a diffuse interest, among different specialists involved in BC management, in widening the boundaries of their knowledge beyond their specialty of origin .

Across-the-board, multidisciplinary training and knowledge are essential for a better and more effective collaboration among specialists, in the patient’s interest. Future breast specialists must have a better understanding of the working environments and competences of their colleagues to optimally apply evidence-based practice to the care of their patients. Treatment in highly specialized multidisciplinary breast centers has been demonstrated to improve quality of life, patients’ satisfaction and chances of survival [1].

According to the survey, despite 64% (*n* = 27) of responders reported having personally changed their clinical practice as a consequence of what they learned through the CCB, only a few (14-33%) were able to implement institutional changes, such as protocols and guidelines update and innovations. Interestingly, though, among these were not only chiefs of department, but also consultants.

As far as networking opportunities and research collaborations, one-third of the participants activated a collaboration with other colleagues, which resulted in two peer-reviewed publications, several new research ideas, and international connections. The majority (27-64%) of the attendees, used the CCB as a trigger to take part in other educational activities, e.g., preceptorships or formally organized Breast Cancer Fellowship programs.

At the Institutional level, only 12% of the participants (*n* = 5) had the opportunity, through the CCB attendance, to visit other BC Units in foreign countries or to be involved in international research projects. These domains were those the participants would like most to be strengthened, despite not being among the CCB objectives.

More than half of the attendees (*n* = 23) profited from attending CCB in terms of their professional career, either in terms of promotions (7-16.7%), change of working institution (4-9.5%) or development of a more structured educational program at their home institutions (12-28.6%).

Despite the results of our survey seem to suggest an overall effectiveness of multidisciplinary specialized training in enhancing the career of professionals dealing with BC, we have to acknowledge that, although all attendees answered the survey, the total number of responders is 42, which is relatively small number to reach a definitive conclusion about the impact of the course program. Together with the lack of a control group, these can be considered the main limitations of our work, affecting the strength of the conclusions.

For the future, the inclusion of more attendees from the third and fourth editions, as well as a comparison with a group of physicians who did not attend the course or have attended other similar programs, may add valuable information to increase the study validity.

The CCB program was previously analyzed by the participants of the second cohort and their bird’s eye evaluation provided useful insights, allowing the CCB scientific chairs to adjust and adapt the modules and the seminars’ content. The current survey, more structured and pondered, is one of the few evaluations available by students attending blended educational programs [18, 19], to our knowledge the only one in oncology.

Despite being conducted in a highly selected and small group of physicians with different cultural, educational, ethnic backgrounds, professionally active in a range of disciplines and working institutions (from university hospitals to private practice, either in western or developing countries), the results provide interesting and stimulating considerations on the expectations and needs of training physicians and the expertise and competence which can be achieved by modern education tools and formats. Both students and teachers need nevertheless to develop and acquire new specific skills to make the most of these evolving educational opportunities [20].

One merit of this survey is to highlight the need to build a breast specialist career and the potential benefits of specialist and multidisciplinary training in breast surgery, which is on average poor and very heterogeneous across Europe, partly because breast surgery is currently just a subspecialty of either general or gynecological surgery.

## Conclusions

Blended learning has the potential to be widely adopted in higher education, prticularly now, due to the COVID-19 pandemic. Learning modalities will undergo a substantial change with a range of positive effects, such as engaging learners, increasing access and flexibility, reducing dropout rates, increasing attendance and satisfaction. Blended learning strategies can make students feel connected with others providing a strong sense of international community in the learning experiences. Another advantage is the affordance of this emerging learning strategy, as reduced travelling and operational costs improve cost-efficiency.

Over pure e-learning, blended courses have the advantage of offering face-to-face sessions which allow for interactions and offer ample opportunities for discussions, enhancing learning outcomes and strengthening the sense of community among trainees and with faculty members.

However, this new instructional strategy presents some pitfalls: technologies could hinder teaching and learning if not used properly, therefore sufficient training and timely technical support should be arranged. Moreover, as individual learning could lead to a lack of motivation, sufficient opportunities for interactions should also be carefully scheduled throughout the whole course, both during in person and online sessions.

The CCB program is one of the few blended programs aiming to build a dedicated, up-to-date CV in BC management. The flexible format, applied also to other ESO certificates of competence, e.g., in lymphoma and lung cancer, may represent a valid model in other oncologic and medical fields, allowing also to overcome current barriers in medical postgraduate training, such as present and future pandemics and funding restrictions.

Understanding the specific strategies and technology use will be critical to improve the CCB course and make the most out of the potentials of blended learning strategy. The optimal balance among face-to-face learning, the proportion of asynchronous or synchronous e-learning, overall duration and suitable evaluation tools, still need to be defined, but the present survey shows that quality and satisfaction are high; timely adjustments can be efficiently planned.

In the absence of a reference standard in postgraduate teaching, it is essential to offer tools that allow trainees to make an accurate and informed choice to build a competitive curriculum according to the needs of modern multidisciplinary oncology. The present survey aims to satisfy this specific need in the field of BC care, recognizing adaptations would be required in other oncology fields.

## Supplementary Information

**Additional file 1.**

**Additional file 2.**

## Data Availability

The final version of the survey and the relative data have been published online on GOOGLE Forms platform and are available from the corresponding author on reasonable request.

## Abbreviations

BC: Breast Cancer
CCB: Certificate of Competence in Breast Cancer
CV: Curriculum Vitae
ESO: European School of Oncology
ECTS: European Credit Transfer and Accumulation System
CAS: Certificate of Advanced Studies
USI: Università della Svizzera Italiana
OECD: Organization for Economic Cooperation and Development
MDM: Multi-Disciplinary Meeting
BRESO: Breast Surgical Oncology Project
