# Supplementary material for: Multidisciplinary blended learning to build a breast cancer specialist career: survey on the perspective of the first 2 cohorts of the ESO-ULM Certificate of Competence in Breast cancer (CCB)
Source: BMC Med Educ. 2022 May 5;22:344. doi: 10.1186/s12909-022-03414-7 (PMC9070614; doi:10.1186/s12909-022-03414-7)
Supplement: Supplementary file 1 — Additional file 1. [file 12909_2022_3414_MOESM1_ESM.docx]

**Multidisciplinary blended learning to build a breast cancer specialist career. A survey on the perspective of the first 2 cohorts of the ESO-ULM Certificate of Competence in Breast Cancer (CCB).**

**Informed Consent**

**Background:**

there is a growing demand for academic education and standardized training among breast cancer specialists to increase their clinical competence in the diagnosis and treatment of patients with breast cancer.

The European School of Oncology (ESO), in co-operation with Ulm University, has developed a structured course, named “Certificate of Competence in Breast Cancer (CCB)”, with the contribution of internationally recognized physicians and scientists in the field of breast cancer.

The Curriculum, focusing on both the clinical and the scientific competence, was established according to several practices and consensus guidelines (European Society for Medical Oncology (ESMO), the San Gallen Breast Cancer Conference, Advanced Breast Cancer ESO-ESMO International Consensus Conference (ABC), ESO-ESMO Breast Cancer in Young Women (BCY) Consensus Conference), with the specific aim of providinge BC specialists with multidisciplinary education.

The CCB has now reached its 3rd edition. 44 specialists in total have been admitted to the first two editions of the Program, completed the course and have satisfactorily passed the final examination. Twenty-five health professionals are currently participating in the 3rd edition.

As a previous participant, we wrote to you inviting you to take part in a survey with the intent to investigate, by means of an online survey, the degree of satisfaction and professional gain, if any, derived from participating to the CCB Program, both on a personal and Institutional level.

Participation in this survey is voluntary.

If you agree to participate, please sign this consent on page 2 and send it back to ESO - Alexandra Zampetti at [azampetti@eso.net](mailto:azampetti@eso.net); you will soon receive the link to an on-line questionnaire. We understand that you have many calls on your time, however, if you can spare some time to complete the survey, we would very much appreciate it.

**Further information:**

your participation is entirely voluntary and by filling the online questionnaire you will consent to take part. You may refuse to take part in the research or exit the survey at any time without penalty or without needing to give a reason. You are free to decline to answer any particular question you do not wish to answer for any reason.

Your responses will be anonymized and analyzed alongside all other responses to produce aggregate results. In line with the open access movement, we are planning to make fully anonymized data publicly available on Google Forms platform for use for research purposes. No identifying information will be contained in this dataset.

If you initially decide to participate but change your mind later, you are free to withdraw by sending an email to the team at azampetti@eso.net. You do not have to provide us with reasons for the termination of your participation. When you withdraw from the study, all your confidential data will be destroyed. If your data has already been analyzed, the results will be used but the source of the data will not be retrievable.

There are no direct personal benefits of participation in this study. However, by participating, you will contribute to the development of better educational tools and formats, tailored on the expectations and needs of training physicians. Results will provide interesting and stimulating considerations for those seeking to build a competitive curriculum according to the needs of modern multidisciplinary oncology.

If you have questions at any time about the study or the procedures, you may contact the principal investigator, Dr. Francesco Meani via email at [francesco.meani@eoc.ch](mailto:francesco.meani@eoc.ch)

We thank you very much for your participation and hope you find the survey enjoyable and thought-provoking.

With best wishes

**Francesco Meani, MD, MS, CEBS**

Clinical Director Centro di Senologia della Svizzera Italiana (CSSI)

via P Capelli 1, 6962 Viganello, Switzerland

*+41 (0)91 811 63 08*

[*francesco.meani@eoc.ch*](mailto:francesco.meani@eoc.ch)

Participant’s Signature

------------------------------------------------

By signing this form, you are consenting to participate in this survey.
