# Supplementary material for: Multidisciplinary blended learning to build a breast cancer specialist career: survey on the perspective of the first 2 cohorts of the ESO-ULM Certificate of Competence in Breast cancer (CCB)
Source: BMC Med Educ. 2022 May 5;22:344. doi: 10.1186/s12909-022-03414-7 (PMC9070614; doi:10.1186/s12909-022-03414-7)
Supplement: Supplementary file 2 — Additional file 2. [file 12909_2022_3414_MOESM2_ESM.pdf]

Multidisciplinary blended learning to build a breast cancer specialist career. A survey on the perspective of the first 2 cohorts of the ESO-ULM Certificate of Competence in Breast Cancer (CCB).

Survey background and rationale

There is a growing demand for academic education and standardized training among breast cancer specialists to increase their clinical competence in the diagnosis and treatment of patients with breast cancer.
The European School of Oncology (ESO), in co-operation with Ulm University, has developed a structured course, named “Certificate of Competence in Breast Cancer (CCB)”, with the contribution of internationally recognized physicians and scientists in the field of breast cancer.
The Curriculum, focusing on both the clinical and the scientific competence, was established according to several practices and consensus guidelines - the European Society for Medical Oncology (ESMO), San Gallen Breast Cancer Conference, Advanced Breast Cancer International Consensus Conference (ABC) and Breast Cancer in Young Women (BCY) with the specific aim to provide BC specialists with multidisciplinary education.
The CCB has now reached its 3rd edition. 42 specialists in total have been admitted to the Program, completed the course and have satisfactorily passed the final examination. Twenty-five health professionals are currently participating in the 3rd edition.
Through the following questionnaire, we would like to investigate the professional gain, if any, derived from participating to the CCB Program, both on a personal and Institutional level.

Please fill the following questionnaire and send it back to Alexandra Zampetti -azampetti@eso.net

1. Background information

Please indicate your country of origin
Country: ……………………………………………………………………………………………………

Please indicate the type of organization in which you were employed at the time of your application and the country
o Regional Hospital
o University Hospital
o Private Practice
o Private Hospital/Clinic
o Other (please specify): ....

Please indicate your country of employment
Country: ……………………………………………………………………………………………………

Please indicate your position at the time of your application
o Post-doc
o Resident
o Fellow
o Ph Student
o Attending physician

o Chief of Department
o Professor
o Other (please specify): ....
Please indicate your field of specialty
o Medical oncology
o Clinical oncology
o Gynecology
o Surgery
o Radiology
o Radiation Oncology
o Plastic Surgery
o Biology
o Nursing
o Other (please specify): ....

Please indicate your main reason to apply to CCB (more than one answers are possible):
o Access to up to date knowledge
o Multidisciplinary education
o Set the basis for new collaborations
o Strengthening personal knowledge in the field
o Develop ideas for research project in the field of BC
o To be added to my CV and boost my career advancement
o Other (please specify): ....

How did you learn about the CCB Program? (more than one answers are possible)
o Own search
o Mail or Newsletter
o ESO Website
o Colleagues
o Head of my department
o Other Institutions
o Previous CCB participant
o Other (please specify): ....

2. Structure of the Program
Please indicate to which extent (1=totally disagree, 6=totally agree) you agree with the following statements regarding information and administration of the program:

Website information was precise and informative	1 2 3 4 5 6
The application procedure was clear and easy to understand	1 2 3 4 5 6
The time spent on the application was acceptable	1 2 3 4 5 6
The fee is in line with the length and complexity of the program	1 2 3 4 5 6

Please indicate to which extent (1=totally disagree, 6=totally agree) you agree with the following statements regarding the evaluation of your application

Processing times were acceptable	1 2 3 4 5 6
Minimum requirements for application were coherent with the program content	1 2 3 4 5 6
Decision criteria for admission were transparent	1 2 3 4 5 6

3. Program design and syllabus

Please indicate to which extent (1=totally disagree, 6=totally agree) you agree with the following statements regarding the CCB Program design:

The program is designed in a way to facilitate opportunities for networking	1 2 3 4 5 6
The program is designed in a way to facilitate implementation of collaborative research projects	1 2 3 4 5 6
The curriculum is well balanced and covers all aspects of multidisciplinary management of breast cancer	1 2 3 4 5 6
Program syllabus is up to date and offers in depth analysis of multidisciplinary BC management	1 2 3 4 5 6
The specific aim of the CCB program is to deliver multidisciplinary knowledge through comprehensive and up-to-date information on all aspects of breast cancer management. Nevertheless it could be advisable, in the future, to structure the CCB curriculum in a way to differentiate, after a common trunk, two separate path according to the applicant's interest in a surgical or oncological career	1 2 3 4 5 6

Please indicate how to improve the CCB program:
o No need for improvements
o Increase the focus on loco-regional management of BC (surgery and radiation oncology)
o Increase the focus on clinical research
o Increase the focus on preclinical research
o Increase practical training during Seminar 3 in Ulm
o Increase multidisciplinary discussion during Seminars
o Include periods of structured observerships, internships or clinical rotations
o Other (please specify): ....
Other (please specify): ....
Other (please specify): ....

4. Program outcome and professional impact

Did the participation to the CCB Program lead to any of the following changes:
Significant changes in your clinical management of BC.
o Yes
o No
o If yes please specify: ……………………………………………………………………..……………………..
If yes please specify: …………………………………………………………………..……………………………..
If yes please specify: …………………………………………………………………..……………………………..

Significant changes in clinical guidelines for BC management at your institution.
o Yes
o No
o If yes please specify: ……………………………………………………………………..……………………..
If yes please specify: …………………………………………………………………..……………………………..
If yes please specify: …………………………………………………………………..……………………………..

Significant changes in the organization of your Breast Center.
o Yes
o No
o If yes please specify: ……………………………………………………………………..……………………...
If yes please specify: …………………………………………………………………..……………………………...
If yes please specify: …………………………………………………………………..……………………………...

Activation of collaborative research projects with other participants of CCB program.
o Yes
o No
o If yes please specify:……………………………………………………………………..…………………
If yes please specify: ………………………………………………………..……………………………
If yes please specify: ………………………………………………………..……………………………

Formal agreements on collaboration between Institutions of other CCB participants.
o Yes
o No
o If yes please specify: ……………………………………………………………………..…………………
If yes please specify: ………………………………………………………..……………………………
If yes please specify: ………………………………………………………..……………………………

Did the participation to the CCB program lead to the following concrete outputs? (more than one answers are possible)
o Publication(s) in international peer reviewed journals
o Publication of books
o Doctoral thesis
o Exchange of students/PhD/attendees
o Any other type of outputs or collaborations.
In this case, please specify ……………………………………………………………..……………..
In this case, please specify ……………………………………………………..……………………..
In this case, please specify ……………………………………………………..……………………..

Please indicate to which extent (1=not relevant, 6=to a great extent) the participation to the CCB Program has led to the following long-term results (impacts)

Multidisciplinary personal knowledge on BC management	1 2 3 4 5 6
Implementation of Multidisciplinary management of BC within your institution	1 2 3 4 5 6
Higher tendency to implement scientific research projects within your institution and or in collaboration with other Institutions	1 2 3 4 5 6
Any other long-term improvements for yourself or your Institution.	1 2 3 4 5 6

Please specify: ....

Did the CCB certificate lead to the following improvements or position changes?
o Change of your position (promotion) within your original Institution
o Change of working institution
o Participation to formally organized and recognized fellowships abroad
o Employment of new professional figures in your Institution
o Implementation of new education programs within your institution
o Participation to other ESO activities/programs
o Any collaboration with Universities:
o Yes
o No
o If yes please specify: ……………………………………………………………………..…………………
If yes please specify: ………………………………………………………..……………………………
If yes please specify: ………………………………………………………..……………………………

Finally give us an overall degree of satisfaction (1=totally disappointed, 6=totally satisfied) about the CCB program 1 2 3 4 5 6

What did you like the most (in a few words)?

....

....

....

What did you dislike the most (in a few words)?

....

....

....

Thank you for your collaboration!
